# Advertising and Young People’s Critical Reasoning Abilities: Systematic Review and Meta-analysis

**DOI:** 10.1542/peds.2022-057780

**Published:** 2022-11-15

**Authors:** Jessica Packer, Helen Croker, Anne-Lise Goddings, Emma J. Boyland, Claire Stansfield, Simon J. Russell, Russell M. Viner

**Affiliations:** a Population, Policy, and Practice Research and Teaching Department, UCL Great Ormond Street Institute of Child Health, University College London, London, United Kingdom; b Department of Psychology, Institute of Population Health, University of Liverpool, Liverpool, United Kingdom; c EPPI-Centre, UCL Social Research Institute, University College London, United Kingdom

## Abstract

**BACKGROUND AND OBJECTIVES:**

Young people are exposed to an abundance of advertising for unhealthy products (eg, unhealthy foods, tobacco, alcohol). Because of their developing cognition, children may not be able to understand the intent of advertising. However, advertising restrictions often assume that adolescents have critical reasoning capacity and can resist the effects of advertising. This review seeks to assess whether the evidence supports this assumption.

**METHODS:**

Ten databases were searched in December 2020. Inclusion criteria were participants aged 6 to 17 years, any advertising exposure, objectively measured understanding or attitudinal outcome, a comparison, control, and between-group comparison. This study included all languages and excluded studies published pre-2010. Two reviewers independently extracted data and assessed study quality.

**RESULTS:**

Thirty-eight articles were included. Meta-analysis of 9 studies with attitudinal outcomes indicated that unhealthy product advertising generated more positive brand or product attitudes compared with neutral or no advertising control in all ages. There were significant effects for digital and nondigital advertising formats. We found greater understanding did not protect against the impact of advertising on brand or product attitudes. Limitations include the inability to meta-analyze the impact of advertising on understanding or the influence of age.

**CONCLUSIONS:**

Evidence shows that the attitudes of young people were influenced by advertising. Critical reasoning abilities did not appear to be fully developed during adolescence and not found to be protective against the impact of advertising. Policymakers should ensure regulations to restrict marketing of unhealthy commodities protects adolescents as well as younger children.

Young people are exposed to an abundance of advertising and marketing, primarily for toys and food products (mostly high in fat, salt, and sugar [HFSS]).^1–4^ Advertising can lead to behavior change through direct and indirect pathways, which leads to harm through unhealthy behaviors.^5^ The hierarchy of effects model suggests that advertising creates awareness of and interest in a brand or product, which leads to heightened preference and then to a decision to purchase and consume.^6^ Much of the advertising children are exposed to is for potentially harmful products (eg, HFSS food, alcohol) which may increase unhealthy behaviors that are associated with a number of detrimental and harmful effects.^7,8^ Direct tobacco advertising is banned in most countries, but young people are still exposed to indirect advertising, for example, through viewing tobacco use on television (TV), shown to result in smoking initiation in young people.^9^ Electronic cigarettes (e-cigarettes) have also grown in popularity over the last decade, and provaping advertising are prevalent on social media, with emerging evidence of harm.^10,11^ Research on the impacts of advertising on children over the past decade has focused particularly on HFSS food advertising.^12^ Young people are exposed to large amounts of food advertising through various media, which is often child-targeted and mostly for HFSS foods.^13,14^ Meta-analyses show that food advertising increases acute calorie intake in children.^15,16^

There has been a strong policy focus on tightening regulations around food advertising, although restrictions frequently only apply to children up to 12 years of age.^17^ There are widespread restrictions to prevent alcohol and tobacco advertising that targets children, since these products are illegal for children to purchase or use,^18^ with calls to make these restrictions worldwide to address noncommunicable diseases.^19^ Researchers have raised concerns over the ability of children and young people to identify, understand, and apply critical reasoning in response to advertising. Thus, they are more susceptible to the influence of advertising, especially in digital formats (including embedded content on webpages, social media platforms and advergames), making this a policy target and active research area.^20^

There is substantial literature on the understanding of advertising. A prominent framework has been the “Persuasion Knowledge Model,” which proposes that to resist advertising, individuals must first recognize that an advert is trying to sell something (persuasion knowledge).^21^ Various aspects of understanding have been identified: recognizing advertising; perception of who pays for advertising and audience targeting; understanding the selling intent of advertising (ie, that advertisers are trying to sell products), persuasive intent (ie, that advertisers are trying to influence behavior via changing attitudes toward products or brands), tactics (ie, specific strategies used), and bias regarding the product (ie, discrepancies between advertised and actual product).^22^ Evidence suggests that “advertising literacy” (ie, knowledge and understanding of advertising intent and tactics) does not fully develop during childhood; therefore, children do not possess the necessary cognitive ability to resist advertising.^22,23^ For this paper, we view critical reasoning as the ability to recognize and understand advertising (advertising literacy) and how it impacts children and young people’s response to advertising. Much of the work around children and advertising, and children’s broader position as consumers, has been informed by Piagetian theory, which presents age-specific stages in children’s development driven by cognitive ability.^24,25^ This suggests progressive growth in understanding, showing that as children get older, cognitive ability increases along with an increased ability to understand and resist advertising. This understanding was largely developed when TV was the main advertising medium, but the applicability to the digital age of advertising has been questioned (even for older children), as entertainment and advertising content are not clearly distinguished.^26^

Social-cognitive models present the effects of advertising occurring automatically without any information processing, suggesting that understanding alone is insufficient to counteract the potentially harmful effects of advertising.^25^ Concerning food, the Food Marketing Defense Model posits that awareness, understanding, ability (including cognitive capacity), and motivation (to resist advertising) are all required to withstand food advertising.^25^ Advertising, especially when digitally embedded, is designed to bypass conscious and rational decision-making and instead rely on emotional responses and unconscious processing, thereby inhibiting the ability to resist effectively.^27,28^

Reasoning abilities are not fully developed by the age of 16, older than the 12-year threshold used in many regulations; other faculties associated with decision-making also continue to develop into adulthood.^29^ It is established that teenagers engage in riskier behavior than both children and adults, attributed in part to changes in reward sensitivity occurring from early adolescence and the later development of self-regulatory competence.^29^ In addition, they may be particularly susceptible to the social influence of their peers.^30^ This evidence may be relevant to young people’s critical reasoning of advertising, since developmentally, they may not be cognitively equipped to protect themselves from the potentially harmful effects of advertising. Studies indicate that children of all ages have difficulties identifying digital marketing.^26,27^ Adolescents are particularly vulnerable to digital advertising because of their engagement with digital technology and media, which plays an important role in their social identity development.^17,27^

Existing reviews and meta-analyses have shown that children of all ages are impacted by advertising,^13–16^ but the notion that understanding of advertising and older age are sufficiently protective remains pervasive. This review focused on 2 areas of interest; the ability of young people to recognize and understand advertising and how they respond to advertising in terms of attitudes toward the advertised brand or product (ie, the impact on diet and attitudes). The review aimed to explore whether evidence supports the notion that critical reasoning ability affects behavioral responses and how this may differ across childhood and adolescence. Critical reasoning relates to the former, but response is likely to include broader factors that could impact on what decisions young people make and their subsequent behavior. For example, attitudes to the advertised product or brand and level of motivation to resist the impact of advertising exposure.

## Methods

We conducted our systematic review using EPPI-Reviewer 4 software.^31^ The study was preregistered with PROSPERO (CRD42018116048), and the systematic review is reported in accordance with the Preferred Reporting Items for Systemic Reviews and Meta-analysis checklist.^32^

### Search Strategy, Eligibility Criteria, and Information Sources

The search strategy was created in collaboration with an information specialist (C.S.). The search was based on terms for population (children and young people), intervention (eg, marketing, advertising, advergame*), and a measure of “understanding” or “attitudes” (eg, reasoning, psychology, advertising literacy, cognition). Systematic searches of the following databases were conducted: ASSIA (Proquest), Child Development and Adolescent Studies (EBSCO), Cochrane Central Database of Controlled Trials, Medline (OVID), PsycINFO (OVID), Sociological Abstracts (Proquest), Social Policy and Practice (OVID) and SCOPUS, and Web of Science – databases (Social Science Citation Index, Emerging Sources Citation Index). The full search strategy is included in a supplemental file (Supplemental Tables 2A, 2B, and 3). Searches were conducted on November 7, 2018 and updated on December 10, 2020. The search results were imported into Endnote reference manager software and duplicates removed. The remaining articles were imported into EPPI-Reviewer 4 software and duplicate records screened and removed; this software was used to manage the screening.

The focus of the review was initially broad, as the scope of the literature was unknown. Following the initial search, a mapping exercise was undertaken to determine the full-text inclusion criteria. A decision was made to focus on experimental studies with an administered exposure (Supplemental Fig 4) for full details of this initial stage and mapping diagram).

Eligible for inclusion during full text screening were: studies with participants aged 6 to 17 years of age inclusive; intervention criteria of any form of advertising for any product (including HFSS products, tobacco, toys); and outcomes of objectively measured understanding (including recognition or identification of advertising, understanding selling, or persuasive intent) or attitudes (toward brand or product including liking or perceptions). Experimental and intervention studies, including randomized or quasi-randomized studies, were included and required to have an appropriate comparison or control group, including no advert, a neutral advert, or a between group comparison (age, gender, socioeconomic status [SES]) with an advert exposure. Neutral adverts were defined by the studies and included adverts that were not the focus of the study, eg, a toy or nonfood advert for studies with a food advertising exposure and food product outcome. Studies were included from 2010 onwards as these were considered most relevant to contemporary advertising practices. There were no restrictions by geography or language. Exclusion criteria were date (pre-2010), intervention (any exposure that evaluated health promotion prevention programs, charity advertising, creation, and testing of models of cognition, media training or advertising literacy, branding only), outcome measures (any nonunderstanding or attitude measures including dietary intake or purchases), study design (qualitative studies, reviews, and dissemination format [nonpeer reviewed], eg, dissertations, conference abstracts, magazine abstracts). A random sample of studies were double-screened by 2 reviewers (H.C., and J.P.) on title and abstract using EPPI-Reviewer 4 software. All screening queries were reconciled by the reviewers. We used the machine learning capabilities of the EPPI-Reviewer software to assist with the screening because of the anticipated number of records from test searches (over 10 000). We employed an “active learning approach,” where the prioritization of records was frequently refreshed so the most relevant articles were screened first. The algorithm was trained using our screening decisions. Articles screened were plotted against studies included, and this was used to indicate when to stop screening (ie, the rate of inclusion plateaued indicating that there were unlikely to be unscreened relevant articles). A classifier model was then created and applied to all unscreened records, with a score based on relevance (0–100) generated and used to double-check exclusion. For full details on the machine learning approach and updated search methods see Supplemental Table 4 and the following reference.^33^ Full-text screening was then independently completed by the same 2 reviewers (H.C. and J.P.) using EPPI-Reviewer 4 software and queries were jointly reconciled.

### Data Extraction

Descriptive data were extracted by 1 reviewer (J.P.) and checked for accuracy by another reviewer (H.C.). Data from experimental studies for inclusion in meta-analyses were independently extracted by 2 authors (J.P., and H.C.) and any discrepancies resolved by reextraction. Corresponding authors were contacted to provide raw data where necessary; 15 authors were contacted for additional information, and 9 provided additional data and 6 did not (1 was contacted regarding understanding outcomes only).

### Assessment of Quality

Risk of bias for the experimental studies was assessed by 2 reviewers (H.C., and J.P.) using Cochrane methods,^34^ either RoB 2.0 for randomized trials^35^ or ROBINS-I tool for nonrandomized studies.^36^ To assess publication bias, funnel plots were created to assess asymmetry using Egger’s test.^37^

### Data Synthesis

For inclusion in meta-analyses for understanding of advertising or brand or product attitudes, studies were required to compare the effect of an unhealthy product (eg, food, alcohol, tobacco) advert exposure to a nonadvert control, or to a control advert (advert for unrelated products).

Studies measuring attitudinal outcomes were required to have mean values with standard deviations. Because of differences in reported outcome measures, which included a variety of different scales (eg, 1–5, 1–3, dichotomous), the DerSimonian-Laird random-effects model was used to allow for synthesis of studies and standardized mean difference (SMD) was used as the outcome for the meta-analyses. All analyses were conducted using Stata 16 (16.1, StataCorp LLC, College Station, TX, USA).^37^ Further details of how advert exposure conditions were combined; the outcome measures and scales and criteria for inclusion in the meta-analyses are provided in a supplemental file (Supplemental Table 5).

Two meta-analyses comparing an advert exposure to control or neutral advert were conducted, by attitude type (brand or product) and by advertising format (digital or nondigital). For this review, we define brand attitude as the attitudes toward the advertised brand and product attitude as the attitudes toward the advertised product. Digital advertising formats included advergames, webpages, social media platforms, and influencer marketing, whereas nondigital advertising formats included TV and printed adverts, and product placement on TV or in movie clips. For all studies except 1,^38^ a single combined advert exposure group was calculated for each group using Cochrane methods.^34^ The exception was a study where 3 separate data points were included with the advert exposure of a specific product matched to the specific product attitudinal outcome measure.^38^ We additionally conducted meta-analyses examining the impact of advertising on attitudes by age (children ≤12 years, teenagers >12 years because of legislation cut-offs).

### Narrative Synthesis

Findings of studies not included in the meta-analysis are reported narratively, presented by outcome (understanding or attitudinal) and by impact of age and advertising features.

## Results

### Study Selection

**FIGURE 1:**
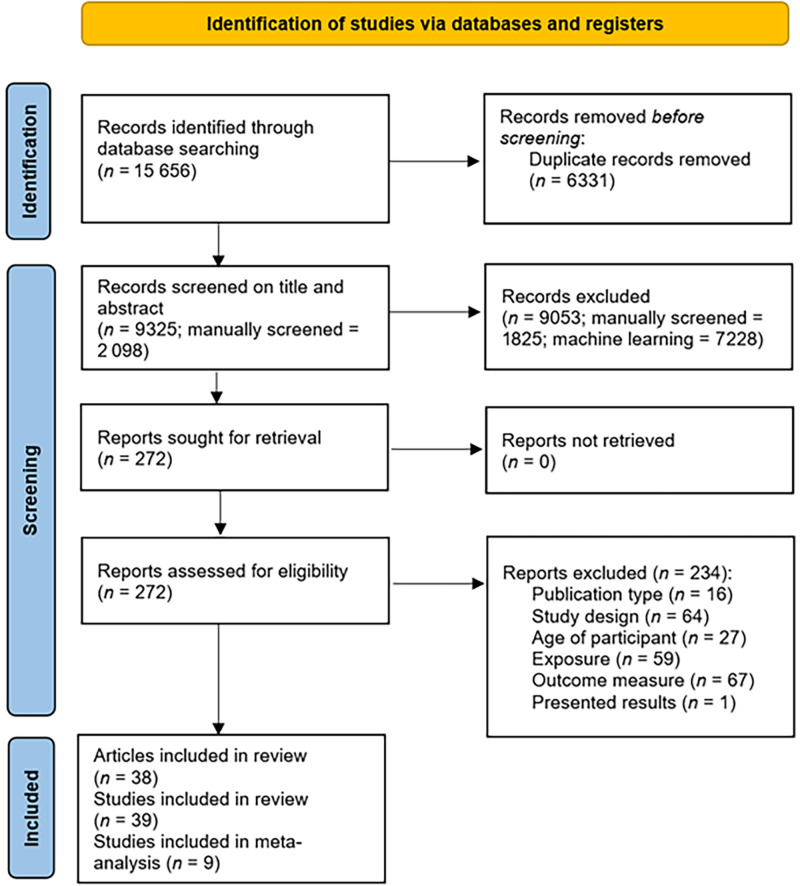
Preferred Reporting Items for Systemic Reviews and Meta-analysis screening flowchart.

The database searches yielded 15 656 papers, resulting in 9325 studies once duplicates were removed. A random subset of 1790 studies were screened on title and abstract to trigger the machine learning from the original search and a further 208 screened on title and abstract from the updated search. Screening on title and abstract ultimately resulted in 272 studies to be screened on full-text and assessed for eligibility. This resulted in 39 studies, from 38 articles, which met the inclusion criteria. Nine of the studies that reported an attitudinal outcome were included in the meta-analyses (Fig 1).

### Study Description and Results

A summary of the descriptive data are provided in Table 1, including details on setting (country, study), participants (sample size, age details), design, advertising exposure, outcomes measures, and findings.

**TABLE 1:** Descriptive Summary of the Included Experimental Studies

Author, Year, Country	Sample Description	Design	Advertising Exposure	Comparison or Control	Outcome	Relevant Results
An,^50^ 2019, South Korea	*n* = 556, age range = 7–11, mean age = NS	Experimental (school), between-subject	3 images of advergame play with HFSS food exposure (Caffé Bene, a national coffee chain, with branded food products = ice cream, sandwiches, bagels, and drinks)	Grade level (age proxy- second and third grade versus fourth and fifth grade)	Ad recognition, skeptical attitudes to advertising	Grade level (age) was a significantly associated to ad recognition and skeptical attitudes, with lower grades (2nd and third) less likely to recognize the advergame as advertising and less likely to have skeptical attitudes, than higher grades (4th and fifth).
Carter,^40^, 2011, Australia	*n* = 594, age range = 4–12, mean age = NS	Experimental (school), between-subject	TV advert for HFSS food brand (McDonald’s)	Age (years)	Selling intent, persuasive intent	All 3 measures of understanding increased significantly with age. Understanding of selling intent was greater than understanding of persuasive intent, which was still only 40% in 11–12 y olds.
Castonguay,^41^ 2015a,^a^ United States, [Study 2]	*n* = 68, age range = 5–11, mean age = NS	Experimental (NS), between-subject	3 TV advert conditions: HFSS cereal (Frosted Flakes); TV Network; computer game. All 30 s adverts placed within 5-min cartoon	Age (5–6 vs 10–11)	Recognition of juxtaposed beliefs	Recognition was significantly greater in older children (10–11 y) compared with younger children (5–6 y).
Castonguay,^51^ 2015b,^a^ United States	*n* = 136, age range = 5–11, mean age = NS	Experimental (research laboratory), within-subject, random assignment	3 TV advert conditions: HFSS cereal (Frosted Flakes); TV network; computer game. All 30 s adverts placed within 5-min cartoon	Age (5–6 vs 10–11)	Product attitude	Younger children in physical activity condition had significantly greater product attitudes than younger without physical activity and both older children groups. The overall difference between combined younger and older groups was not tested.
Dias and Agante,^42^ 2011, Portugal	*n* = 231, age range = 7–8, mean age = NS	Experimental (school), between-subject, random assignment	Advergame for HFSS products (ice cream, potato chips, cookies, soda, pizza, lollipop, hamburger + chocolate mousse), 5-min play in groups of 1–4	Noncommercial game with healthy products (fruit, vegetables, milk, bread)	Food liking, nutritional knowledge	Children exposed to the advergame had significantly higher preference for the HFSS products compared with those exposed to the non-commercial game. No impact on nutritional knowledge.
Dixon,^43^ 2017, Australia	*n* = 904, age range = 5–9, mean age = 7	Experimental (school), between-subject, random assignment	TV adverts for HFSS food (McDonald’s) shown after a 30-s movie trailer with movie tie in premium for (1) unhealthy meals (2) healthy meals or (3) both	Neutral TV advert (leisure activity)	Meal preference, product perceptions	Significantly higher preference for healthier meal if only healthy meals had movie tie in premium compared with other conditions. Significantly more positive perceptions when the healthier meals had movie tie in premium, compared with having none.
Duke,^54^ 2016,^b^ United States	*n* = 3665, age range = 13–17, mean age = NS	Experimental (online), between-subject, random assignment	4×TV adverts for e-cigarettes (3 × 60-s and 1 × 30-s)	No advert	E-cigarette attitudes	Significantly more positive e-cigarette attitudes in the treatment condition compared with control.
Farrelly,^55^ 2015,^b^ United States	*n* = 3665, age range = 13–17, mean age = NS	Experimental (online), between-subject, random assignment	4×TV adverts for e-cigarettes (3 × 60-s and 1 × 30-s)	No advert	Perceived benefits of e-cigarettes	TV ads positively and significantly impacted on e-cigarette beliefs compared with control.
Harris,^44^ 2018, United States	*n* = 138, age range = 7–11, mean age = 9.4	Experimental (research center), between-subject, random assignment	TV adverts for HFSS food (Ribena drink, Kellogg’s choc snack bar, McVitie’s biscuits) with a healthy message (health halo) or nonhealth message or healthy products (milk, pistachios, cheesestrings)	Age (7–9 vs 10–11)	Perceived risks and benefits	Age was not a predictor. Health halo advert condition perceived nutrient poor products as significantly healthier than the other 2 conditions.
Hudders and Cauberghe, ^45^ 2018, Belgium	*n* = 180 age range = 7–12, mean age = 8.69	Experimental (school), between-subject	TV advert (90-s) for Wii within a movie excerpt (4-min cartoon, Alvin and the Chipmunks: The Squeakquel)	Age (7–9 vs 10–12)	Identification of commercial content, advertising, literacy brand attitude (interaction only)	Identification of commercial content and advertising literacy was significantly greater in older children compared with younger children. Found advertising literacy was not significantly related to brand attitude.
Kim,^56^ 2017, United States	*n* = 802, age range = 13–17, mean age = NS	Experimental (online), between-subject, random assignment	3 x TV adverts for e-cigarettes from a pool of 14 (30-s to 2-min)	3×neutral TV adverts (bottled water) from a pool of 7 (30-s to 2-min)	Perceived risks and benefits	In never-smokers only, perceived risk of cigarettes was significantly lower in intervention compared with control.
Lapierre,^46^ 2015, United States	*n* = 79, age range = 6–9, mean age = 7.7	Experimental (Camp, afterschool center), between-subject	3 × 30-s TV adverts (2 for toys and 1 for HFSS cereal- HoneyNut Cheerios)	Age (years)	Persuasive intent, selling intent	Age was not a significant predictor of understanding measures. Understanding of selling intent was significantly higher than persuasive intent.
Matthes and Naderer,^61^ 2015, Austria	*n* = 121, age range = 6–14, mean age = NS	Experimental (school), between subject, random assignment	Product placement for HFSS food (UTZ Cheese Balls) within 7-min movie excerpt (cartoon, Alvin and the Chipmunks) with moderate or high frequency product placement	No advert control, age (years)	Brand attitude, product attitude	No effect of placements on brand or product attitudes, compared with control. Brand attitude decreased with age.
Naderer,^62^ 2016, Austria	*n* = 109, age range = 8–13, mean age = 10.76	Experimental (school), between-subject, random assignment	Advergame (Visa branded Monopoly), approximately 30 min play time	Unbranded game (Monopoly), approximately 30 min play time, age (years)	Brand attitude	Brand attitude was significantly higher in the advergame condition compared with no advert control. Age was not a significant predictor.
Naderer,^74^ 2018, Austria	*n* = 363, age range = 6–15, mean age = 10.55	Experimental (school), between-subject, random assignment	Product placement of HFSS product (M&Ms) in movie (7-min clip of Smurfs) with static placement (shown in background) or character product involvement (interacts with the product)	Control, 7-min clip of Smurfs with no product placement	Brand evaluation	No difference in brand evaluation between the placement conditions or no advert control.
Neyens,^63^ 2017, United Kingdom	*n* = 940, age range = 6–14, mean age = 9.8	Experimental (school), between-subject, random assignment	Advergame for HFSS food (Kellogg’s Coco-Pops, 10-min play time) OR TV advert for HFSS food (Kellogg’s Coco-Pops, 19-s embedded within 10-min TV clip for youth series)	No advert control, age (years)	Persuasion knowledge, brand attitude, brand preference	Persuasion knowledge was significantly higher for the TV ad versus advergame. Children who played the advergame reported significantly more positive brand attitudes compared with children who had watched the TV ad and children in the no advertising exposure control group. Age was significantly positively related to persuasion knowledge and negatively related to brand attitude.
Owen,^47^ 2013, United Kingdom	*n* = 134, age range = 6–10, mean age = NS	Experimental (school), between- subject	Shown 2/5 following HFSS adverts: (1) brand placement in movie (Dr Pepper in Spiderman), (2) TV sponsorship (Cadbury chocolates in Coronation Street), (3) product licensing (Shrek on Nestlé cereal), (4) advergame (McDonald’s Web site), (5) in-game product placement (Red Bull energy drink on PlayStation 2 game)	Age (6–7 vs 9–10)	Understanding of advertising	Understanding of advertising was significantly higher among older children compared with younger. Understanding of TV advertising was significantly greater than non-traditional advertising in all children.
Padon,^57^ 2018, United States	*n* = 417, age range = 13–17, mean age = 15	Experimental (online), between-subject, random assignment	4 x TV adverts for e-cigarettes either low or high youth appeal (each less than 30 s)	4×neutral TV adverts (food or drink)	Product attitude, product beliefs	Positive product beliefs increased significantly in low youth appeal ads compared with control, high youth appeals increased positive product beliefs, but it was not significant.
Panic,^53^ 2013, Belgium, [Study 2]	*n* = 128, age range = 7–10, mean age = 8	Experimental (school), between-subject, random assignment	Advergame for HFSS food (Lay’s crisps, 2-min play)	Noncommercial game (healthy food- fruit and vegetables)	Persuasion knowledge	No significant differences in persuasion knowledge between the commercial and non-commercial advergames.
Petrescu,^64^ 2017, United States	*n* = 411, age range = 11–16, mean age = 13.09	Experimental (home), between-subject, random assignment	10×printed advert for e-cigarettes either glamourized or associated with health	No advert control	Appeal of smoking or e-cigarettes, prevalence estimates, perceived attributes of smoking, perceived harms of smoking	Significantly increased estimation in prevalence of e-cigarette use in glamour condition compared with control and health condition. In both experimental conditions perceived danger and harm of occasional smoking were rated lower than control.
Rifon,^48^ 2014, United States	*n* = 376, age range = 5–10, mean age = 7.3	Experimental (test site), between-subject, random assignment	Advergame for HFSS food (Honey O’s cereal) that children played or watched (designed to mimic tv ad) and with brand integrated in game or shown in background (play time determined by child)	Unbranded game, age (5–7 vs 8–10)	Persuasion knowledge, brand attitude, perceived healthiness, taste expectations	Persuasion knowledge increased with age, playing exposure and brand integration. Integrated brand conditions had increased taste expectations, perceived healthiness, but this was moderated by play and age. Treatment conditions had significantly more positive taste expectations in treatment compared with control.
Royne,^38^ 2017, United States	*n* = 64, age range = 6–11, mean age = NS	Experimental (research facility), between-subject, random assignment	Product placement for cola, juice or milk embedded in TV cartoon (SpongeBob SquarePants, 15-min clip)	No product placement control (same 15-min TV clip	Product liking, perceived healthiness	For “likes juice” outcome, all treatments conditions had significantly higher results than control. For the “perceived healthiness of juice” outcome the milk and cola conditions were significantly greater than control. No other results were significant.
Sharma,^65^ 2015, India	*n* = 1050, age range = 10–17, mean age = NS	Experimental (NS), between-subject, random assignment	Printed advert for HFSS food (biscuit) OR mobile handset with picture, caricature or product information	Age (10–12 vs 13–17)	Brand attitude	Teenagers had significantly lower brand attitude toward biscuits in the model's picture and product information settings compared with tweenagers.
Smith,^52^ 2020, Australia	*n* = 156, age range = 7–12, mean age =	Experimental (university), between-subject, random assignment	3 advert conditions for HFSS product (unfamiliar confectionery): (1) banner advertisement, (2) advergame (4-min play time), (3) rewarded video advertisement	Control group with no advertising	Brand perception, awareness of advertising	Across groups there were no significant differences between pr- and postgame ratings of taste or fun. Awareness of advertising was highest in rewarded video advertising condition 80% (only significant finding) then, advergame condition 60%, compared with just 31% of participants in the banner advertisement condition.
Tarabashkina, ^66^ 2016,^c^ Australia	*n* = 354, age range = 7–13, mean age = NS	Experimental (agricultural event), between-subject, random assignment	Pop-up advert for HFSS food (biscuit) within a 10-min internet exposure	Neutral pop-up advert (toy) within a 10-min internet exposure, age (7–8 vs 9–10 vs 11–12 vs 13)	Selling intent, persuasive intent, product evaluation, nutritional knowledge	No differences in cluster membership based on age, including selling and persuasive intent, product evaluation and nutritional knowledge. A trend toward choosing the advertised product was seen in the experimental group compared with control but was not significant.
Tarabashkina, ^67^ 2018a,^c^ Australia	*n* = 326, age range = 8–13, mean age = NS	Experimental (agricultural event), between-subject	Poster advert on a bus stop for a fictitious HFSS food product (burger)	Age (8–9 vs 10–11 vs 12–13)	Informative intention, product liking intention, attention capturing intention, persuasion attribution of the advertisement	There were no significant differences in any perceived advertising intention variables by age group.
Tarabashkina, ^68^ 2018b,^c^ Australia	*n* = 175, age range = 7–13, mean age = NS	Experimental (agricultural event), between-subject	Online pop-up advert for HFSS food (cookie) shown 3 times during a 10-min internet search session (2nd, fifth and eighth minute)	Age (7–8 vs 9–10 vs 11–13)	Perceived informative intent, perceived affective intent, persuasive intent, product preference, product taste, product healthiness	There were no significant differences in any of the variables by age group, except for product healthiness which the oldest age group rated as significantly lower compared with the youngest age group. Higher persuasive intent understanding led to decreased favorable food preference and lower healthiness evaluation.
Te'eni-Harari, ^69^ 2014, Israel	*n* = 252, age range = 4–15, mean age = 9.45 (3.24)	Experimental (school), between-subject	TV advert for 4 fictitious products named “ZOZO” HFSS food (hot dog), phone, book, or toothpaste (each 20-s)	Age (4–7 vs 8–11 vs 12–15)	Brand attitude	Age had a significantly negative effect on brand attitude.
Uribe and Fuentes-García,^70^ 2015,^d^ Chile	*n* = 483, age range = 9–15, mean age = NS	Experimental (school), between-subject, random assignment	3 advert conditions for HFSS brand (McDonald’s) embedded in movie clip (Richie Rich, 45-min): (1) product placement (2 scenes), (2) 2 x TV adverts, (3) 1×product placement and 1 x TV advert	No advert or product placement, same 45-min film (Richie Rich) Age (9 vs 12 vs 15)	Brand attitude	There were no significant differences in brand attitude between any of the treatment or age groups.
Uribe and Fuentes-García, ^75^ 2020,^d^ Chile	*n* = 376, age range = 9–15, mean age = 12	Experimental (school), between-subject, random assignment	Product placement for HFSS product (McDonald’s) embedded in movie clip (Richie Rich, 45-min, 2 scenes)	Age (9 vs 12 vs 15)	Recognition of the commercial nature of the message, brand preference	Recognition of advertising significantly increased as the age of the children increased (9 vs 12 vs 15). Brand preference significantly decreased as age of the children increased (9 vs 12 vs 15).
van Berlo,^58^ 2017,^e^ Netherlands	*n* = 73, age range = 13–18, mean age = 15.48	Experimental (school), between-subject, random assignment	Advergame (making pizzas) with an unknown or well-known pizza brand	Unbranded game (making pizzas)	Advertising wisdom, brand attitude	No significant differences in brand attitude between the conditions.
van Berlo,^59^ 2020,^e^ Netherlands	*n* = 98, age range = 13–18, mean age = 14.95	Experimental (school), between-subject, random assignment	4-min advergame play with HFSS food (making pizza): familiar brand (Domino’s) and unfamiliar brand (Nonna’s pizza)	4-min game play with HFSS food (making pizza) with no brand logo	Recognition of commercial intent, brand attitude (unfamiliar and familiar brand)	Recognition of commercial intent in the familiar brand condition was significantly greater than game without a brand. No difference in recognition between familiar or unfamiliar brands or unfamiliar brand and no brand condition. There were no differences in brand attitude toward the familiar or unfamiliar brands between any of the conditions.
van Reijmersdal, ^71^ 2010, Netherlands	*n* = 2453, age range = 10–17, mean age = 12.68	Experimental (online, at home), between-subject, random assignment	Advergame play (“GoSupermodel”) with product placement (Dutch bank) time determined by child	No game play control or noncommercial game play (time determined by child)	Brand image	Brand image results were significantly greater in the advergame play condition.
van Reijmersdal, ^76^ 2020, Netherlands	*n* = 406, age range = 12–16, mean age = 14	Experimental (school), between-subject, random assignment	YouTube video with well-known YouTuber sponsored by HFSS product (Fanta)	Age (12–14 vs 15–16)	Recognition of sponsored content as advertising, understanding persuasive intent	In the no disclosure group, 12–14 y olds had significantly higher recognition of sponsored content as being advertising compared with 15–16-y-olds. No significant difference between age groups for understanding persuasive intent.
Vasiljevic,^72^ 2018, United Kingdom	*n* = 1449, age range = 11–16, mean age = NS	Experimental (school), between-subject, random assignment	10×printed glamorous e-cigarette advert	Neutral advert (nonsmoking related)	Perceived harm of occasional and regular use, prevalence estimates of e-cigarettes and cigarettes	Children exposed to glamorous e-cigarette adverts perceived the harms of occasional smoking of 1 or 2 tobacco cigarettes to be lower than those in the control group. No significant differences between the experimental conditions for perceived harm of or prevalence estimates for e-cigarettes or cigarettes.
Verhellen,^73^ 2014, Belgium	*n* = 125, age range = 11–14, mean age = 11.98	Experimental (school), between-subject, random assignment	4 advert conditions for HFSS food (Ola popsicles): (1) traditional TV ad, (2) trailer, (3) advergame, (4) trailer + advergame	No advert control	Persuasion knowledge, brand attitude	No significant differences in brand attitude or persuasion knowledge between the experimental conditions. Children without persuasion knowledge developed a significantly more positive attitude toward the brand than children with persuasion knowledge.
Vogel,^60^ 2020, US	*n* = 135, age range = 13–18, mean age = 15.3	Experimental (online), between-subject, random assignment	Instagram advert posts for e-cigarettes with heavy e-cigarette content (3 e-cigarette posts and 3 unrelated posts) or light e-cigarette content (1 e-cigarette posts and 5 unrelated posts)	No advert control, shown peer generated posts for e-cigarettes with heavy or light e-cigarette content	Attitudes about using e-cigarettes, risk perceptions of e-cigarettes	Participants in advert source condition had significantly greater positive attitudes toward e cigarettes, compared with peer generated source. No difference in perceived risks between sources conditions. No difference in perceived risks between e-cig conditions.
Waiguny,^49^ 2014, Austria, [Study 1]	*n* = 51, age range = 8–10, mean age = NS	Experimental (school), between-subject, random assignment	2 advert conditions for HFSS food (Nesquik Duo, cereal): (1) advergame (7:24-min play time), (2) TV advert (30-s)	Age (year)	Persuasion knowledge, identification of commercial content	No effect of age on the measure of persuasion knowledge or identification of commercial content. Greater identification of commercial content in TV advert compared with advergame.
Waiguny,^49^ 2014, Austria, [Study 2]	*n* = 149, age range = 7–10, mean age = NS	Experimental (school), between-subject	Advergame for HFSS food (Nesquik Duo, cereal, 10-min play time),	No advert control	Persuasion knowledge, identification of commercial content, brand beliefs, brand reference	Advergame exposure significantly positively influenced children’s brand beliefs and preferences, compared with control. Difference in persuasion knowledge between the conditions was not separately assessed. Identification of commercial content was generally higher with a higher level of persuasion knowledge but was negatively overridden by presence in the game.

NS, not stated.

a Half of the sample may be reported in both.

b Same sample but reporting of different outcomes.

c May be the same participants across all 3 studies.

d May be the same participants across the 2 studies.

e Three out of the 4 schools may be reported in both.

Participant ages ranged from 4 to 18 and were broadly categorized as 12 years and under (*n* = 19),^38,39–52^ over 12 years (*n* = 7),^53–59^ or had participants in both age groups (*n* = 13).^60–75^ Most of the studies were conducted in Europe (*n* = 16; Austria *n* = 5, Netherlands *n* = 4, Belgium *n* = 3, UK *n* = 3, Portugal *n* = 1), followed by the United States (*n* = 12), Australia (*n* = 6), Chile (*n* = 2), and Israel (*n* = 1), India (*n* = 1), South Korea (*n* = 1). Studies were mostly conducted in classroom settings (*n* = 21). Advertising exposure was most commonly for food (*n* = 29; all included a HFSS product or brand, eg, fast food or sugary cereal; in addition to some non-HFSS products), followed by e-cigarettes (*n* = 7) or an assortment of products (*n* = 3, including games, banks, and a financial services company). Majority of the advertising exposures were nondigital (*n* = 25, including TV adverts, product placement, print advert, TV sponsorship, or movie trailers), compared with digital (*n* = 18, including advergames, banner or pop-ups, social media). Outcomes, related to the advertised product, measured either understanding (*n* = 10, eg, identification of commercial content, selling intent, persuasive intent, perceived advertising intentions) or attitudinal (*n* = 23, eg, product liking, product perceptions, perceived benefits, appeal) or studies that measured both (*n* = 13).

### Narrative Synthesis of Understanding Outcomes

Meta-analysis was not possible for understanding measures, owing to the heterogeneity of exposures and outcomes for relevant studies. Many studies had control groups where, because of the nature of the questions, understanding of advertising was not able to be assessed (ie, cannot assess understanding about an advert the group did not see).

### Impact of Age on Understanding

Where compared across age groups, understanding of advertising increased significantly with age (8 studies, mostly assessed as some concerns of bias and 1 as low risk of bias),^39,40,41,46,47,49,62,74^ although no significant effects were found in 4 studies (mostly assessed as having some concerns of bias and one as low risk of bias),^45,48,66,67^ and understanding decreased with age (assessed as low risk of bias).^75^ Most of these studies were conducted with children under 12 years, so evidence was limited for teenagers. Of 2 studies conducted with teenagers, 1 study assessed as having some concerns of bias directly compared children aged 9, 12, and 15 years and found that advertising recognition significantly increased as age increased^74^; the other study assessed as low risk of bias found 12 to 14 years olds had significantly higher recognition of sponsored content in a YouTube video compared with 15 to 16 year olds, but there was no significant difference between age groups for understanding persuasive intent.^75^

### Impact of Advert Content on Understanding

One study with some concerns of bias reported that persuasion knowledge increased with higher brand integration (in relation to advergames), but persuasion knowledge was very low across all groups and the magnitude of differences modest.^47^ In relation to child “involvement” with advertising (ie, engagement with advergame), 1 study with some concerns of bias showed that children more involved with an advergame were less likely to identify commercial content.^48^ One study with low risk of bias looked at differences in recognition of commercial content in advergames between a familiar HFSS brand and a fictitious or unbranded pizza game and found that recognition of the familiar brand was significantly greater than the unbranded game.^58^ A similar study with some concerns of bias assessed persuasion knowledge between a branded advergame and a noncommercial advergame and found no significant difference.^52^ Seven studies of mixed bias assessments (4 with some concerns, 3 low risk) measured different types of understanding; 4 found that awareness of selling intent was higher than persuasive intent in children aged 4 to 12 years (2 were significant^39,45^; 2 did not test significance)^46,48,62^; 2 found recognition of advertising in 7 to 16 year olds was greater than understanding persuasive intent^76^ or advertising literacy^44^; finally, 1 found skeptical attitudes toward advertising were greater than recognition of advergames as advertising, because of very low recognition in 7 to 11 year olds (62.5% to 72% vs 48.5%).^49^ Four studies with some concerns of bias measured the impact of advertising format and found significantly greater understanding with nondigital advertising (TV) compared with digital advertising (primarily advergames).^46,48,62^ Overall, understanding of the persuasive intent of adverts to impact on attitudes and behaviors was generally low across studies, for example, only 40% in 11 to 12 year olds,^39^ and only 1% of 7 to 9 year olds and 12% 10 to 12 year olds.^44^

### Meta-analyses of Attitudinal Outcomes

**FIGURE 2:**
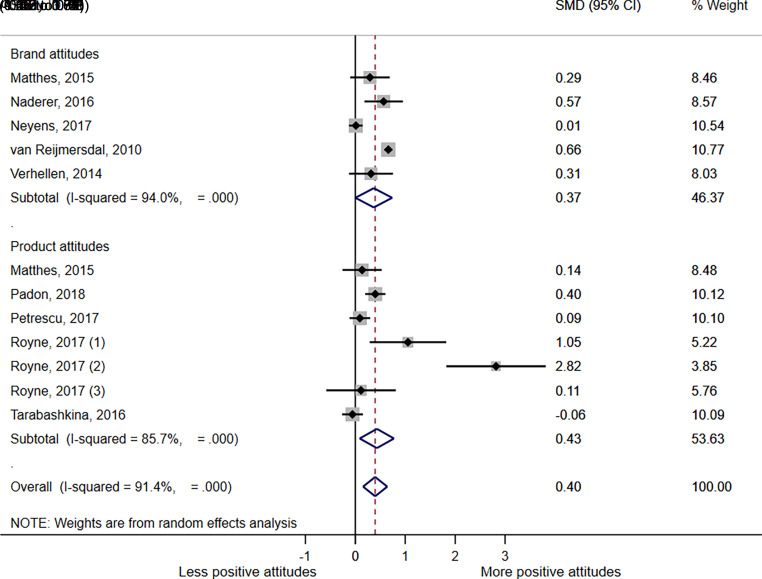
Forest plot showing SMD in brand and product attitudes between any advertising exposure and no advert or neutral advert controls; 95% CIs and study weights are indicated. Overall SMD was generated by a random effects model. (1) Data from cola product placement vs control with cola attitude question; (2) Data from juice product placement vs control with juice attitude question; (3) Data from milk product placement vs control with milk attitude question.

A meta-analysis comparing all advert exposures to no advert or neutral advert control by attitude type (Fig 2), showed that overall, any advertising exposure significantly increased positive attitudes toward the brand or product, SMD = 0.397 (*P* = .001; 95% confidence interval [CI] 0.154–0.639; I^2^ = 91.4%). The subgroup meta-analysis by attitude type also showed that the effect of an advertising exposure was significant for both product attitudes (SMD = 0.430 [*P* = .014; 95% CI 0.087–0.774; I^2^ = 85.7%]) and brand attitudes (SMD = 0.369 [*P* = .049; 95% CI 0.001–0.736; I^2^ = 94.0%]).

**FIGURE 3:**
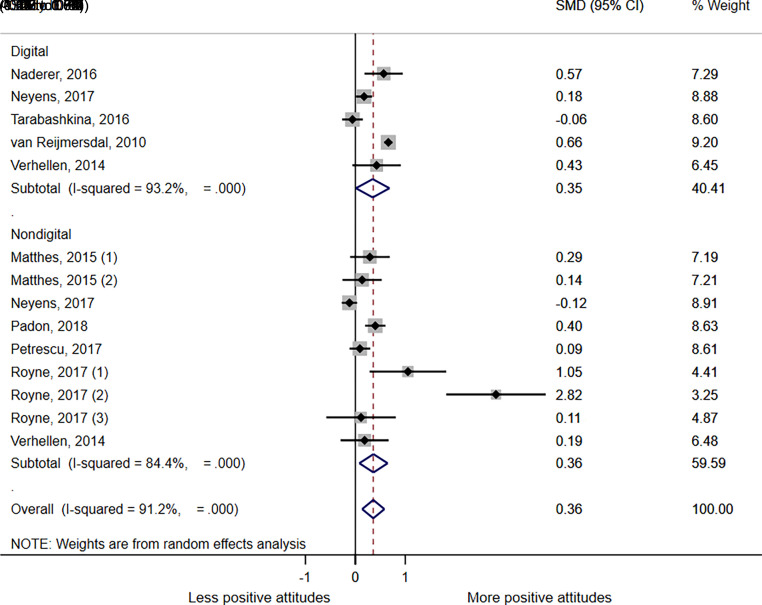
Forest plot showing SMD in brand or product attitudes between digital and non-digital advertising exposure and no advert or neutral advert controls; 95% CIs and study weights are indicated. Overall SMD was generated by a random effects model. Matthes (1) brand attitude outcome; Matthes, (2) product attitude outcome; Royne (1) data from cola product placement versus control with cola attitude question; Royne (2) Data from juice product placement versus control with juice attitude question; Royne (3) data from milk product placement versus control with milk attitude question.

A meta-analysis exploring the effect of advertising by format (Fig 3) showed overall that any advert significantly increased positive attitudes, compared with no advert or neutral control, SMD = 0.36 (*P* = .009; 95% CI 0.14–0.58; I^2^ = 91.2%). When examined by advertising format, both digital advertising exposure and non-digital advert exposures had a significant positive effect on attitudes, SMD = 0.35 (*P* = .005; 95% CI 0.01–0.068; I^2^ = 93.2%) and SMD = 0.36 (*P* = .005; 95% CI 0.08–0.65; I^2^ = 84.5%), respectively. Egger’s regression analysis found no evidence of bias for either meta-analysis, although funnel plots showed some evidence of asymmetry (Supplemental Figs 5 and 6). Trim and fill analysis showed no strong evidence of missing studies for either meta-analysis (Supplemental Figs 7 and 8). Sensitivity analysis was completed running a fixed effect model; none of the findings changed in significance in either direction.

An additional meta-analysis was conducted, which looked at the impact of advertising on attitudes by age (Supplemental Fig 9). Advertising had a positive impact on attitudes compared with the control condition for both age groups (ie, >12 years and ≤12 years). A further meta-analysis was carried out as a sensitivity analysis (Supplemental Fig 10) to explore whether the effect held when the largest effect size was removed and the effect was still seen.

### Narrative Synthesis of Attitudinal Outcomes

The majority of controlled studies not suitable for meta-analysis supported the above findings, namely that adverts brought about more positive attitudes (7 studies, mixed bias assessments: 3 low, 3 some concerns and 1 high)^41,42,47,48,53,57,59^; however, 5 studies found no significant differences between groups (mixed bias assessment: 3 low, 2 some concerns).^51,57,58,69,73^ One study, assessed as having a high risk of bias, explored the impact of e-cigarette adverts designed with low and high youth appeal and found the low youth appeal advert resulted in more positive attitudes than a none-cigarette control advert, but there was no difference between the high youth appeal and control adverts.^56^ One study, assessed as low risk of bias, found that the younger group (5–6 years) had significantly more positive product attitudes following exposure to TV advert for HFSS cereal compared with the older group (10–11 years).^50^ Another study with some concerns of bias found that brand preference following exposure to product placement decreased significantly with increasing age (9 vs 12 vs 15 years).^74^

Two studies with low risk of bias examined the impact of glamorized e-cigarette advertising on perceptions of cigarette smoking or e-cigarettes, compared with neutral or no advert control. They found the adverts led to occasional cigarette smoking being perceived as less dangerous and harmful^63,71^ and the use of e-cigarettes by children as being more common.^63^ One also found there was no difference in the appeal of e-cigarettes between adverts that glamorized e-cigarettes compared with adverts that associated e-cigarettes with health.^63^

### Impact of Understanding on Attitudinal Outcomes

Seven studies measured the interaction between understanding and attitudinal outcomes and reported interactions. Five studies found no interaction, showing that greater understanding of advertising did not limit favorable attitudes toward the advertised product^44,47,48,58,65^ and 2 found some evidence of an interaction.^62,67^ Six of these studies were found to have some concern of bias, and the other was assessed to have low risk of bias.^58^ This study found, for children aged 13–18 years, recognition of commercial intent had no effect on brand attitude for either an unfamiliar or familiar brand. The age range for children from studies that found no interaction was broader than those that found interactions (5–18 vs 7–14 years). Of the 2 studies that found an interaction between lack of persuasion knowledge and greater attitudinal outcomes, the first had online pop-up adverts, which are heavily embedded, as the advertising exposure,^72^ whereas the second only found an interaction among children that understood the snack was unhealthy (the interaction was not observed if children thought the advertised snack was healthy).^67^

### Quality Assessment

For nonrandomized studies, 2 were rated as low and 11 as moderate risk of bias (Supplemental Fig 11). Moderate risk of bias was mostly caused by the domain “bias caused by confounding,” as not enough information was provided or confounding variables were not included in analyses. Of the randomized studies, 10 were rated as low risk of bias, 13 as some concerns, and 3 with high risk of bias (Supplemental Fig 12). The studies with some concerns were mostly because of lack of detail about the randomization process or unreported information about the selection of the reported results. Results were consistent between studies rated as low to high risk of bias. Sensitivity analyses were run excluding studies rated as high risk from the meta-analysis (Supplemental Fig 14). The overall impact of advertising on attitudes remained but product attitude subgroup was no longer significant.

## Discussion

In this systematic review, data suggested that children’s understanding of advertising intent was limited and not nuanced, ie, children could recognize that adverts intended to sell a product but not that these were intended to change their attitudes and behavior. There was limited evidence that understanding increased with age, but more research is needed in this area. Understanding was lower for digital compared with nondigital formats, and lower when children were more involved with the medium (eg, advergames or online advertising). In terms of attitudes, meta-analyses indicated that advertising brought about more positive attitudes to both brands and products compared with controls; this was observed across all age groups. There was no evidence that adverts with high “youth appeal” were more effective, but evidence was limited for these exposures. Findings suggested that greater understanding of advertising is not protective, with evidence that attitudinal outcomes were impacted positively regardless of level of understanding. These findings collectively indicate that advertising impacts children, regardless of age, level of understanding, format, or specific targeting or youth appeal.

Our findings indicate that children and young people of all ages have some difficulties in understanding advertising. This fits with the developmental perspective that young people’s critical reasoning abilities continue developing into late adolescence.^29^ We found that greater understanding does not necessarily protect against advertising, consistent with the Food Marketing Defense Model that challenges the focus on understanding to counteract the effects of advertising. The model instead proposes that advertising influences young people without conscious processing and that motivation to resist is also required, which may be lower among young people.^25^ We did not include disclosure or media literacy intervention exposures in this review, but our findings suggest that the inclusion of disclosures (eg, declarations stating “this is an advert”) or media literacy training designed to increase understanding or advertising literacy would not necessarily protect children and adolescents from the influence of advertising.^76^ This is supported in the literature as 1 experiment found that children who viewed food marketing with a disclosure actually consumed significantly more of a marketed snack than a control group.^76^ A study in adolescents found that disclosures did not mitigate persuasion and increased brand memory, despite increasing understanding of persuasive intent.^77^ Media literacy programs are a strategy often suggested by the food and beverage industry to increase persuasion knowledge in children, in lieu of improved regulations, such as industry-funded Media Smart (see https://mediasmart.uk.com/).^78,79^

Our findings that advertising had a positive impact on attitudes are consistent with previous research on food advertising.^12,14,80,81^ Further supporting these findings, adverts (TV and advergames) for “unhealthy” unfamiliar food products have been found to elicit positive attitudes in children (aged 7–12 years) to a greater extent with advergames compared with TV advertising.^82^ We found effects on attitudes regardless of age, consistent with other studies in different age groups. There is evidence that preschool children exposed to adverts for a range of child-directed foods had positive attitudes about these foods,^83^ and that adolescents reported positive attitudes after viewing online adverts for fast food and confectionery.^84^

Comparing digital and nondigital advertising formats, we found no difference in impact on attitudes in subgroup meta-analysis, but narrative synthesis indicated that understanding was lower for digital formats. This is unsurprising since digital advertising is more integrated and, therefore, may be less explicit and more difficult to identify and understand, in addition to greater personalization and targeting.^21,25^ This is important given the ubiquity of these formats, especially for adolescents, who because of their extensive engagement with digital media with less supervision, may be more susceptible to digital advertising.^85^ For adolescents, media plays an important role in their social identity development, as they place more value on the opinions and actions of peers and figure out their perception of how they fit with others.^17,18,27^ Digital marketing, especially on social media, is designed to target these unique developmental vulnerabilities.^86^

### Implications

The findings from this review support understanding not being fully developed during childhood or adolescence. We also found that advertising influences the attitudes of young people of all ages, suggesting a need to protect older as well as younger children. Our results suggest that understanding does not protect children from the harmful impacts and influence of advertising, as per the Food Marketing Defense Model.^25^ Reducing exposure to advertising is therefore likely to be more effective than improving understanding through disclosures or media literacy training. Existing regulations typically only apply to children up to 12 years of age, as they have historically been regarded as more vulnerable to advertising, therefore needing greater protection.^87^ Our findings do not support lesser restrictions for advertising to teenagers, as there is no distinct evidence-based threshold for understanding that supports a cut-off of 12 years and suggest that appropriate protection from advertising exposure would benefit all young people.^17^

### Limitations

The limitations of this review include a lack of suitable data or studies to meta-analyze the impact of advertising on understanding or the influence of age. Meta-analysis limitations include the high heterogeneity of studies, despite using a random effects model and standardized mean difference outcome. The machine learning method has limitations, as a large number of articles were excluded without screening on title and abstract. The majority of the included studies were assessed as having some concerns of bias, which needs to be taken into consideration when interpreting the findings, although sensitivity analyses removing studies with high risk of bias and the largest effect size were conducted and not found to impact results. We may not have identified all product placement exposure studies, as this term was not included in our search strategy; however, studies with advert or marketing key words were included. Some of the studies may have been conducted in the same or similar group of participants (Tarabashkina^66–68^; Duke and Farrelly^53,54^; Uribe^69,74^; van Berlo^57,58^; Castonguay^40,50^), but these do not interfere with the meta-analysis as only 1 was included. The time since the searches were completed is a limitation, with original searches completed in October 2018 and then updated in December 2020. This subject area is complex, so the review process is time intensive. Updating the searches would be low yield as the substantive findings of the work remained unchanged following the update searches, and we have no reason to believe the main findings of the paper would be subject to change. The main strength of the paper is that it meets an evidence gap, specifically addressing if children over 12 years of age have critical reasoning capacity and can therefore resist the effects of advertising. We were also able to quantitatively assess the impact of advertising on attitudinal outcomes. The search was carefully planned and executed, with double screening and data extraction. Studies were contemporary, adding to the relevance for current policy. Because of the delay observed in research, we found fewer studies using digital advertisement exposures, which is an area where more primary research is needed. There is also a need for further primary research in teenagers in relation to critical reasoning and advertising, and especially digital formats.

## Conclusions

This systematic review and meta-analysis provide evidence that advertising impacts upon the attitudes of children and young people of all ages, regardless of their level of understanding and critical reasoning abilities. These findings may be useful to inform the thinking of policy makers, particularly in terms of restrictions based on age and changing patterns of media consumption.

## Supplementary Material

Supplemental Information

## Abbreviations

CI: confidence interval
e-cigarettes: electronic cigarettes
HFSS: high in fat, salt, and sugar
SES: socioeconomic status
SMD: standardized mean difference
TV: television
