# Supplementary material for: Advertising and Young People’s Critical Reasoning Abilities: Systematic Review and Meta-analysis
Source: Pediatrics. 2022 Nov 15;150(6):e2022057780. doi: 10.1542/peds.2022-057780 (PMC9724173; doi:10.1542/peds.2022-057780)
Supplement: Supplemental Information [file PEDS_2022057780SupplementaryData.pdf]

Supplemental Information

Articles excluded during mapping stage (n = 301)

- Brain imaging (n = 11)
- Year (n = 108)
- Public service announcement (n = 77)
- Media training (n = 27)
- Branding (n = 26)
- Self esteem/ body image (n = 22)
- Qualitative (n = 19)
- Models (n = 7)
- Scale or tool development (n = 1)
- Charity (n = 3)

SUPPLEMENTAL FIGURE 4
Mapping exercise details and diagram. The search was purposively inclusive, as the scope of the literature was largely unknown. The initial inclusion criteria were studies of any design (including experimental, intervention, cross-sectional, longitudinal, qualitative) with participants aged 6 to 17 years of age (inclusive), an advertising exposure for any product (eg, TV advertisement, advergame) and a measure of "understanding" (eg, understanding of advertising intent, recognition of advertising) or "attitudes" (eg, liking of the brand or product advertised). Since the literature identified in the-search was extensive and heterogenous (531 studies were identified as potentially relevant on title and abstract from the original search), a mapping exercise was undertaken to narrow the literature to best address the study aims (Supplemental Fig 4). Following consultation with the wider research team, experimental studies with an administered exposure were chosen as the focus, to manage the large number of heterogenous studies. This yielded 272 studies eligible for full text screening.

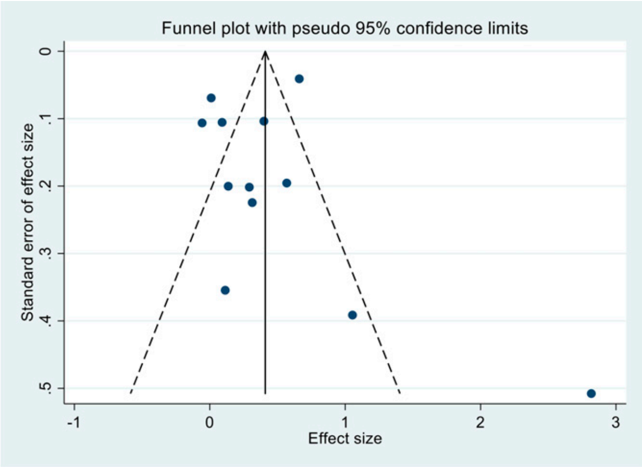

SUPPLEMENTAL FIGURE 5
Funnel plot for brand and product attitudes meta-analysis (corresponds to meta-analysis presented in Figure 2).

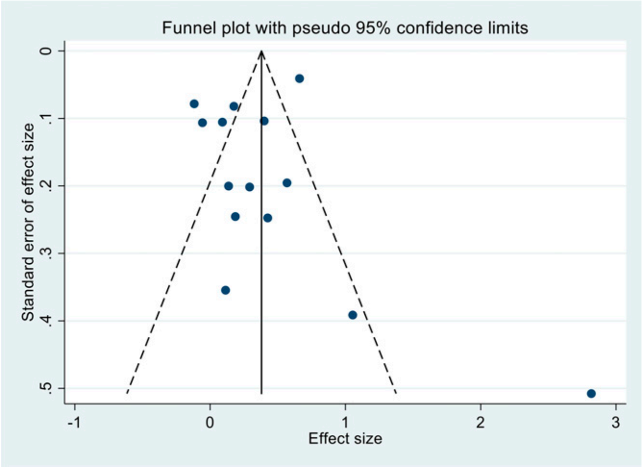

SUPPLEMENTAL FIGURE 6
Funnel plots for digital versus non-digital meta-analysis (corresponds to meta-analysis presented in Figure 3).

```
. metatrim _ES _seES, egger

Note: default data input format (theta, se_theta) assumed.

Meta-analysis

       |  Pooled      95% CI         Asymptotic      No. of
Method |     Est   Lower   Upper  z_value  p_value   studies
-------+----------------------------------------------------
Fixed  |   0.410   0.353   0.467   14.150    .000       12
Random |   0.397   0.154   0.639    3.199    .001

Test for heterogeneity: Q= 128.334 on 11 degrees of freedom (p =.000)
Moment-based estimate of between studies variance =  0.141

Trimming estimator: Linear
Meta-analysis type: Fixed-effects model

iteration |  estimate    Tn    # to trim     diff
----------+--------------------------------------
    1     |     0.410     32         0          78
    2     |     0.410     32         0           0

Note: no trimming performed; data unchanged

Filled
Meta-analysis

       |  Pooled      95% CI         Asymptotic      No. of
Method |     Est   Lower   Upper  z_value  p_value   studies
-------+----------------------------------------------------
Fixed  |   0.410   0.353   0.467   14.150    .000       12
Random |   0.397   0.154   0.639    3.199    .001

Test for heterogeneity: Q= 128.334 on 11 degrees of freedom (p= .000)
Moment-based estimate of between studies variance =  0.141
```

SUPPLEMENTAL FIGURE 7
Trim and fill analysis for brand and product attitudes meta-analysis (corresponds to meta-analysis presented in Figure 2).

```
. metatrim _ES _seES, egger

Note: default data input format (theta, se_theta) assumed.

Meta-analysis

       |  Pooled      95% CI         Asymptotic      No. of
Method |     Est   Lower   Upper  z_value  p_value   studies
-------+----------------------------------------------------
Fixed  |   0.380   0.326   0.434   13.745    .000       14
Random |   0.359   0.136   0.581    3.163    .002

Test for heterogeneity: Q= 148.006 on 13 degrees of freedom (p =.000)
Moment-based estimate of between studies variance =  0.138

Trimming estimator: Linear
Meta-analysis type: Fixed-effects model

iteration |  estimate    Tn    # to trim     diff
----------+--------------------------------------
    1     |    0.380     43        0         105
    2     |    0.380     43        0           0

Note: no trimming performed; data unchanged

Filled
Meta-analysis

       |  Pooled      95% CI         Asymptotic      No. of
Method |     Est   Lower   Upper  z_value  p_value   studies
-------+----------------------------------------------------
Fixed  |   0.380   0.326   0.434   13.745    .000       14
Random |   0.359   0.136   0.581    3.163    .002

Test for heterogeneity: Q= 148.006 on 13 degrees of freedom (p =.000)
Moment-based estimate of between studies variance =  0.138
```

SUPPLEMENTAL FIGURE 8

Trim and fill analysis for digital versus nondigital meta-analysis (corresponds to meta-analysis presented in Figure 3).

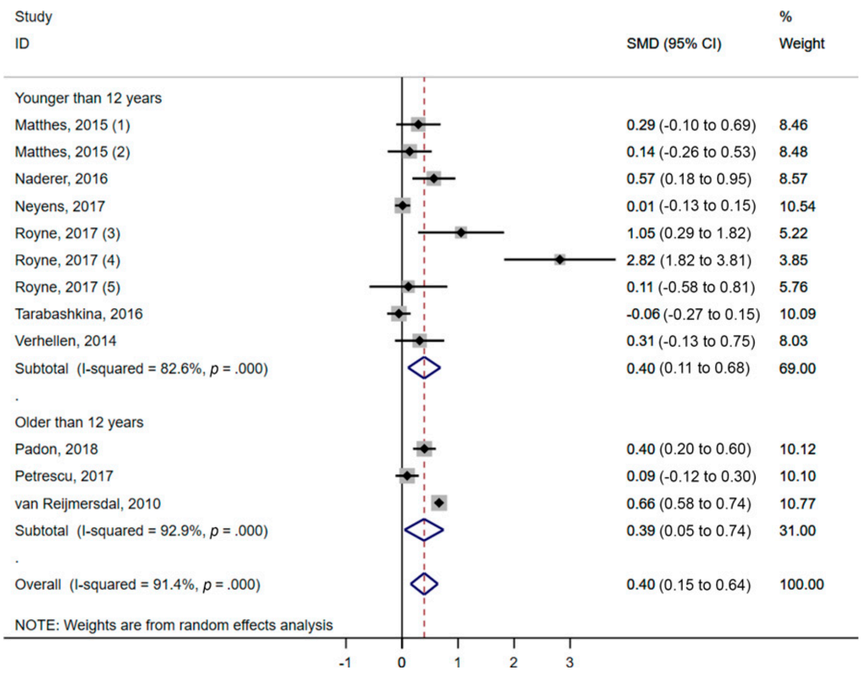

SUPPLEMENTAL FIGURE 9

Forest plot showing SMD in brand or product attitudes between any advertising exposure and no advert or neutral advert controls by age of participants (mean age under or over 12 years); 95% CIs and study weights are indicated. Overall, SMD was generated by a random effects model. (1) Brand attitude outcome, (2) product attitude outcome, (3) data from cola product placement versus control with cola attitude question, (4) data from juice product placement vs control with juice attitude question, (5) data from milk product placement versus control with milk attitude question.

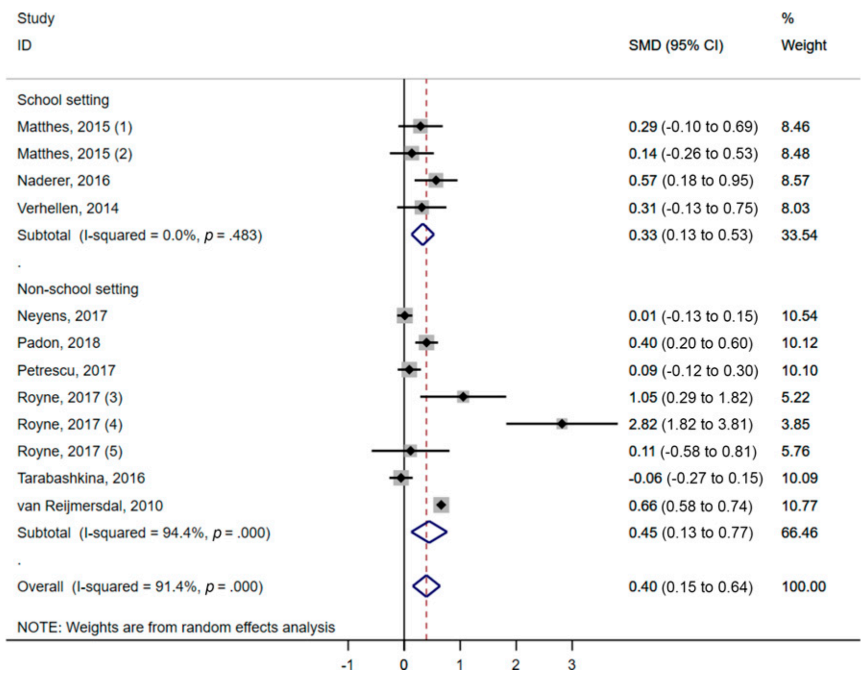

SUPPLEMENTAL FIGURE 10

Forest plot showing SMD in brand or product attitudes between any advertising exposure and no advert or neutral advert controls by experiment setting, school or nonschool; 95% CIs and study weights are indicated. Overall, SMD was generated by a random effects model. (1) Brand attitude outcome, (2) product attitude outcome, (3) data from cola product placement versus control with cola attitude question, (4) data from juice product placement versus control with juice attitude question, (5) data from milk product placement vs control with milk attitude question.

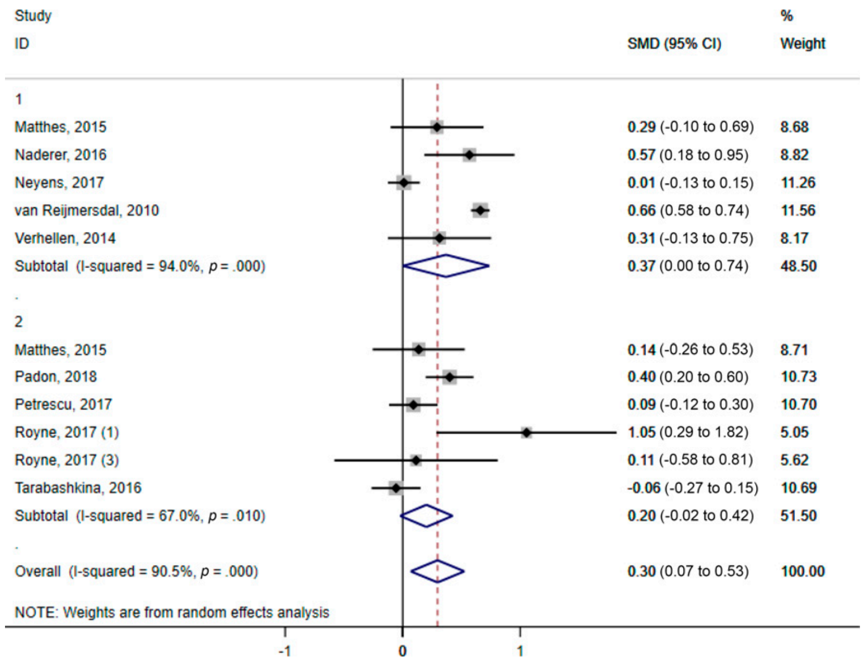

SUPPLEMENTAL FIGURE 11

Sensitivity analysis – excluding largest effect size MA1: Forest plot showing SMD in brand or product attitudes between digital and non-digital advertising exposure and no advert or neutral advert controls; 95% CIs and study weights are indicated. Overall, SMD was generated by a random effects model.

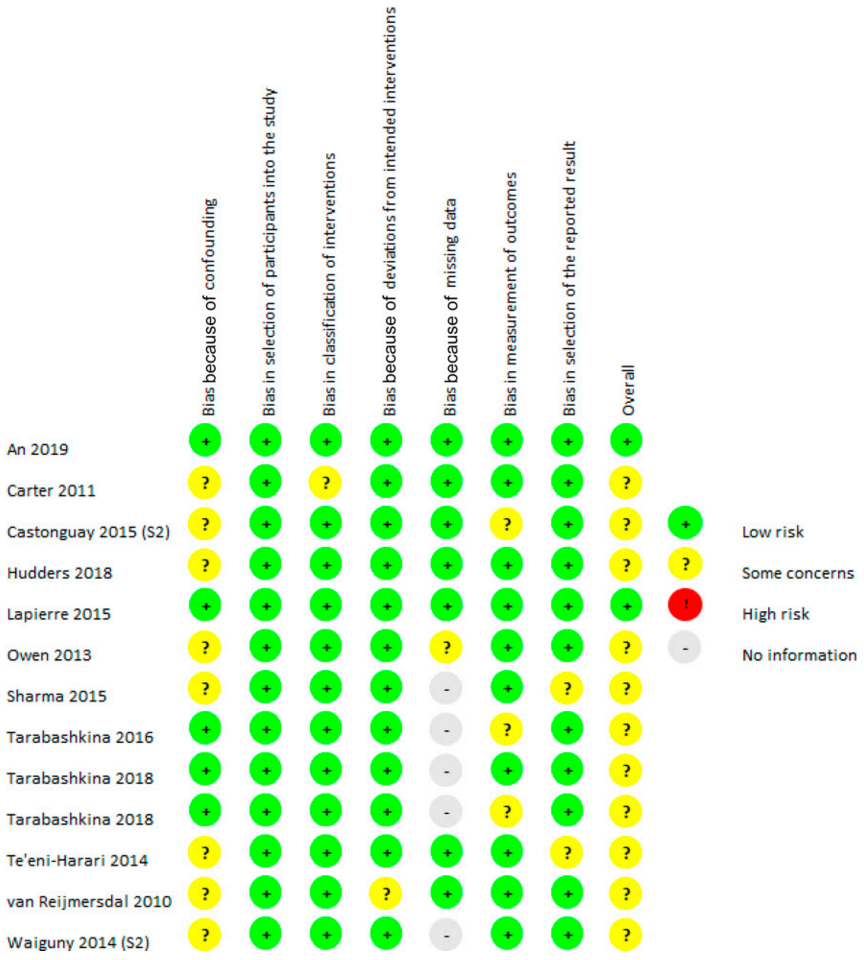

SUPPLEMENTAL FIGURE 12

Bias assessment for nonrandomized experimental studies using ROBINS-I.

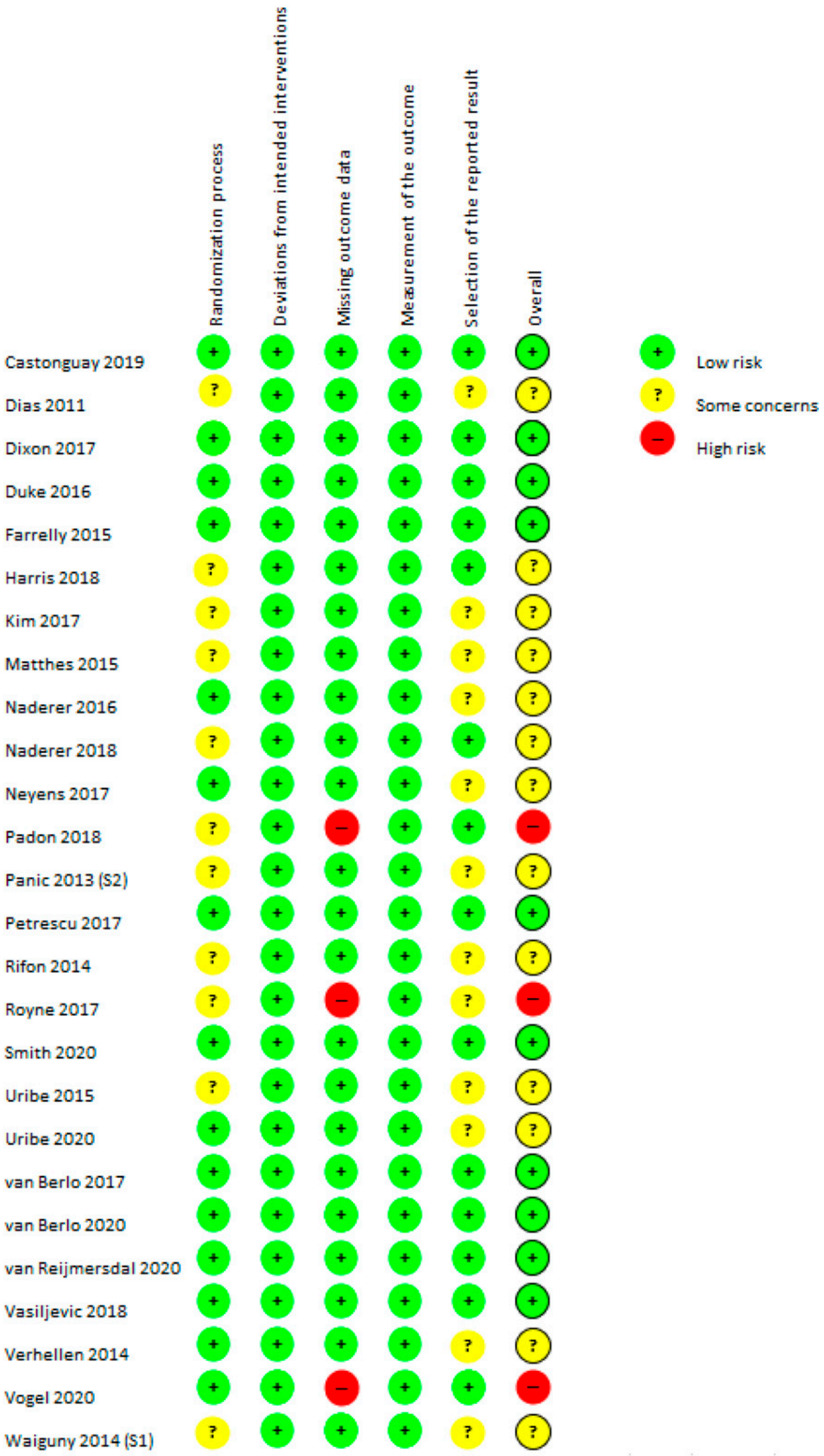

SUPPLEMENTAL FIGURE 13
Bias assessment for randomized experimental studies using RoB-2.

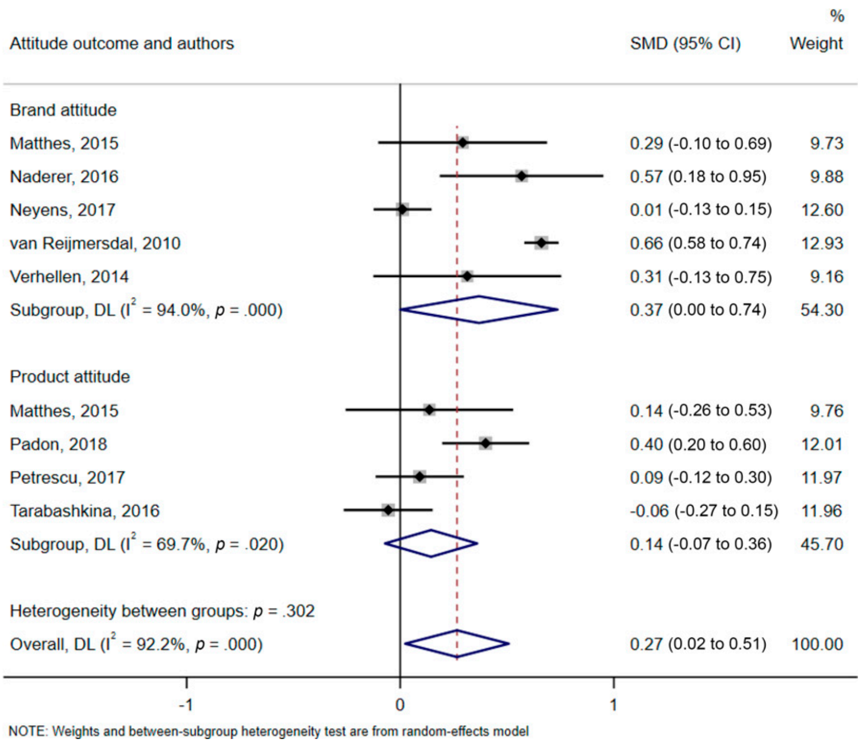

SUPPLEMENTAL FIGURE 14

Sensitivity analysis – excluding all Royne studies from MA1: forest plot showing SMD in brand or product attitudes between digital and nondigital advertising exposure and no advert or neutral advert controls; 95% CIs and study weights are indicated. Overall, SMD was generated by a random effects model.

SUPPLEMENTAL TABLE 2A Details of Search and Search Terms

	Concept 1 Participants	Concept 2 Exposure	Concept 3 Outcomes
Cochrane and Medline	Adolescent (13–18y), child (6–12y)	“Advertising as topic” (marketing N/A from definitions)	Comprehension, judgment, decision making, cognition, thinking, psychology, child, psychology, adolescent
PsycInfo	NA	Advertising	Comprehension, judgment, decision making, cognitive processes, adolescent attitudes, child attitudes
ProQuest (ASSIA)	Children, young people, adolescents	Advertising and advertisements	Comprehension, decision making, cognitive processes
Keywords	child*, adolescen*, youth*, young person* or people* schoolchild*, “school child*”, boy*, girl*, teen*, school*child (NA for every database)	advert*, marketing, advergam*, commercials, “television commercial*”, “TV commercial*”, “radio commercial*”, “media commercial*”	judgement*, judgment*, attitud*, cogniti*, reasoning, media literacy, advertising literacy, appraisal*, recognition*, psychology, decision*, understanding*, belief*, perception*, comprehension*

NA, not applicable. Databases searched include Ovid Medline, Cochrane, Scopus, Psych Info, ProQuest (Central)- ASSIA, Web of Science- social science and emerging sources, Social Policy and Practice, and Child Development and Adolescent Studies.
* refers to a wildcard symbol used for truncations in the searches.

SUPPLEMENTAL TABLE 2B Screening Criteria

Participants	6–17y (to distinguish from preschool and adult samples)
Intervention (exposure)	Adverts (any form, eg, TV, advergames, online, poster)
Comparison	Experimental and intervention studies: advert versus nonadvert; food advert versus nonfood advert (including group comparisons where data available- age, gender, SES); between-group comparisons (age, gender, SES) with advert exposure (ie, no control group); if the literature is very limited, we could include within-child changes (ie, change over the intervention or experiment in the sample)- but would be difficult to interpret; real-world studies: cross-sectional; between-group comparisons (age, gender, SES); between-group comparisons with different levels of advert exposure; subgroups combining these (ie, different levels of advert exposure and different age, gender, or SES groups); real-world studies: longitudinal; between-group comparisons (age, gender, SES) over time; between-group comparisons over time with different levels of advert exposure at baseline; subgroups combining these (ie, different levels of advert exposure and different age, gender, and SES groups); within-child comparisons over time in a sample (no breakdown by characteristics or advert exposure)- again suggest doing this only if other literature is limited
Outcome measure	Must have some measure of judgement; we will additionally record other more distal outcomes (eg, behavior, opinion)
Study designs	experimental; intervention; “real-world” (cross-sectional or longitudinal)
Other	
Geography	All
Languages	All
Time	All until 09/12/2020

SUPPLEMENTAL TABLE 3 Search History

Database	Search Terms
Ovid Medline(R) and epub ahead of print, in-process, and other non-indexed citations and daily	(1) exp child/ (2) exp adolescent/ (3) (child* or adolescen* or youth* or young person* or young people* or schoolchild* or boy* or girl* or teen* or school child*).ti,ab,id. (4) 1 or 2 or 3 (5) exp ADVERTISING AS TOPIC/ (6) (advert* or marketing or advergam* or commercials or TV commercial* or television commercial* or radio commercial* or media commercial*).ti,ab,id. (7) 5 or 6 (8) exp COMPREHENSION/ (9) exp JUDGMENT/ (10) exp Decision Making/ (11) exp cognition/ (12) exp thinking/ (13) exp psychology, child/ (14) exp psychology, adolescent/ (15) (judgement* or judgment* or attitud* or cogniti* or reasoning or advertising literacy or media literacy or apprais* or recognition* or psychology or decision* or understanding* or belief* or comprehension* or perception*).ti,ab,id. (16) 8 or 9 or 10 or 11 or 12 or 13 or 14 or 15 (17) 4 and 7 and 16
PsycINFO	(1) exp advertising/ (2) (child* or adolescen* or youth* or "young person*" or "young people*" or schoolchild* or boy* or girl* or teen* or "school child*").ti,ab,id. (3) (advert* or marketing or advergam* or commercials or "TV commercial*" or "television commercial*" or "radio commercial*" or "media commercial*").ti,ab,id. (4) (judgement* or judgment* or attitud* or cogniti* or reasoning or "media literacy" or "advertising literacy" or apprais* or recognition* or psychology or decision* or understanding* or belief* or comprehension* or perception*).ti,ab,id. (5) exp JUDGMENT/ (6) exp comprehension/ (7) exp Decision Making/ (8) exp Cognitive Processes/ (9) exp adolescent attitudes/ (10) exp child attitudes/ (11) 1 or 3 (12) 4 or 5 or 6 or 7 or 8 or 9 or 10 (13) 2 and 11 and 12 (14) 11 and 12 (15) limit 14 to (180 school age <age 6 to 12 y> or 200 adolescence <age 13 to 17 y>) (16) 13 or 15
Cochrane ID Search	(1) MeSH descriptor: [Child] explode all trees (2) (child* OR adolescen* OR youth* OR (young NEXT person*) OR (young NEXT people*) OR schoolchild* OR boy* OR girl* OR teen* OR school child*).ti,ab,id. (3) MeSH descriptor: [Adolescent] explode all trees (4) MeSH descriptor: [Advertising as Topic] explode all trees (5) (advert* or marketing or advergam* or commercials or TV commercial* or television commercial* or radio commercial* or media commercial*).ti,ab,id. (6) MeSH descriptor: [Comprehension] explode all trees (7) MeSH descriptor: [Judgment] explode all trees (8) MeSH descriptor: [Decision Making] explode all trees (9) MeSH descriptor: [Cognition] explode all trees (10) MeSH descriptor: [Thinking] explode all trees (11) MeSH descriptor: [Psychology, Child] explode all trees (12) MeSH descriptor: [Psychology, Adolescent] explode all trees (13) (judgement* or judgment* or attitud* or cogniti* or reasoning or advertising literacy or media literacy or apprais* or recognition* or psychology or decision* or understanding* or belief* or comprehension* or perception*).ti,ab,id. (14) #1 OR #2 OR #3 (15) #4 OR #5 (16) #6 OR #7 OR #8 OR #9 OR #10 OR #11 OR #12 OR #13 (17) #14 AND #15 AND #16
Scopus	(TITLE-ABS(child* OR adolescen* OR youth* OR (young W/1 person) OR (young W/1 people) OR schoolchild* OR boy OR girl OR teens OR teenager OR {school-child} OR {school-children}) OR AUTHKEY(child* OR adolescen* OR youth* OR (young W/1 person) OR (young W/1 people) OR schoolchild* OR boy OR girl OR teens OR teenager OR {school-child} OR {school-children})) AND (TITLE-ABS(advert* OR advergam* OR {commercials} OR {TV commercial} OR {television commercial} OR {radio commercial} OR {media commercial} OR {marketing}) OR AUTHKEY(advert* OR advergam* OR {commercials} OR {TV commercial} OR {television commercial} OR {radio commercial} OR {media commercial})) AND (TITLE-ABS(judgement* OR judgment* OR attitud* OR cogniti* OR reasoning OR {media literacy} OR {advertising literacy} OR apprais* OR recognition* OR "psychology" OR decision* OR understanding* OR belief* OR comprehension* OR perception*) OR AUTHKEY(judgement* OR judgment* OR attitud* OR cogniti* OR reasoning OR {media literacy} OR {advertising literacy} OR apprais* OR recognition* OR "psychology" OR decision* OR understanding* OR belief* OR comprehension* OR perception*))
ProQuest (ASSIA)	((MAINSUBJECT.EXACT.EXPLODE("Children") OR MAINSUBJECT.EXACT.EXPLODE("Young people") OR MAINSUBJECT.EXACT.EXPLODE("Adolescents") OR TI,AB(child* OR adolescen* OR youth* OR "young person*" OR "young people*" OR schoolchild* OR boy* OR girl* OR teen* OR "school*child*")) AND (MAINSUBJECT.EXACT.EXPLODE("Advertising") OR MAINSUBJECT.EXACT.EXPLODE("Advertisements") OR TI,AB(advert* OR marketing OR advergam* OR commercials OR "TV commercial*" OR "television commercial*" OR "radio commercial*" OR "media commercial*")) AND (MAINSUBJECT.EXACT.EXPLODE("Comprehension") OR MAINSUBJECT.EXACT.EXPLODE("Decision making") OR MAINSUBJECT.EXACT.EXPLODE("Cognitive processes") OR TI,AB(judgement* OR judgment* OR attitud* OR cogniti* OR reasoning OR "media literacy" OR "advertising literacy" OR apprais* OR recognition* OR psychology OR decision* OR understanding* OR belief* OR comprehension* OR perception*)))
Web of Science	(TS=(child* OR adolescen* OR youth* OR "young person*" OR "young people*" OR "schoolchild*" OR boy* OR girl* OR teen* OR "school*child*")) AND (TS= (advert* OR "marketing" OR advergam* OR "commercials" OR "TV commercial*" OR "television commercial*" OR "radio commercial*" OR "media commercial*")) AND (TS= (judgement* OR judgment* OR attitud* OR cogniti* OR "reasoning" OR "media literacy" OR "advertising literacy" OR apprais* OR recognition* OR "psychology" OR decision* OR understanding* OR belief* OR comprehension* OR perception*))<br>Timespan:All years.Indexes:SSCI, ESCI.

SUPPLEMENTAL TABLE 3 Continued

Database	Search Terms
Social Policy and Practice	1. ((child* or adolescen* or youth* or "young person*" or "young people*" or schoolchild* or boy* or girl* or teen* or "school-child*") and (advert* or marketing or advergam* or commercials or "TV commercial*" or "television commercial*" or "radio commercial*" or "media commercial*")).ab,ti,nt,de,hw. and (judgement* or judgment* or attitud* or cogniti* or reasoning or "media literacy" or "advertising literacy" or apprais* or recognition* or psychology or decision* or understanding* or belief* or comprehension* or perception*).ti,ab,nt,de,hw.
CDAS	(child* or adolescen* or youth* or "young person*" or "young people*" or schoolchild* or boy* or girl* or teen* or "school*child*") AND (advert* OR marketing OR advergam* OR commercials OR "TV commercial*" OR "television commercial*" OR "radio commercial*" OR "media commercial*") AND (judgement* OR judgment* OR attitud* OR cogniti* OR reasoning OR "media literacy" OR "advertising literacy" OR apprais* OR recognition* OR psychology OR decision* OR understanding* OR belief* OR comprehension* OR perception*)

SUPPLEMENTAL TABLE 4 Details About Machine Learning

Relevance scores for each study based on a random sample of studies that had been manually screened were generated using the review management software, with higher scores indicating greater relevancy. Duplicate screening on the highest relevancy scores continued manually until 6 irrelevant studies in a row were screened (score = 47). All studies with this relevance score or higher were screened and all studies with lower scores were excluded (a random sample of excluded studies below this threshold were checked, $n = 50$). For the updated search, the classifier model was applied to the new studies (once duplicates were removed) and relevancy scores generated. The same cut-off score was applied, with studies above that score included for full-text screening and studies below that score excluded (a random sample of excluded studies was screened, $n = 50$).

SUPPLEMENTAL TABLE 5 Rationale for Meta-analysis Inclusion and Data Processing

Author, year	Data	Outcome measure	Scale	Comparison
Matthes,[61] 2015	Y	Brand attitude - children were shown a picture of the UTZ brand logo and asked whether they found the picture "likeable" and "funny." Both items were combined. Product attitude - children were asked whether they found UTZ Cheese Balls "likeable" and "funny."	0–2, 0–2	Control (same movie clip with no placement) versus combined experimental (mod or high freq)
Naderer,[62] 2016	Y	Brand attitude -"preference for Visa" - children were requested to: evaluate the Visa logo; pick which of the 3 credit card-brands they liked the most; and which 1 they would prefer to use in the future. Three items were on the same scale 0 = I like it not at all to 3 = I like it very much, combined.	0–3	Control versus branded intervention
Neyens,[63] 2017	Y	Brand attitude - asked to indicate how much they liked the brand on a 1-item, 5-point Likert smiley-scale ranging from "not at all" to "very much"	1–5	Control (no advert) versus combined experimental condition (advergame or TV)
Padon,[57] 2018	Y	Product beliefs - participants reported their agreement on 5-point Likert scales ranging from strongly disagree to strongly agree with the following items, "E-cigarettes are … cool, enjoyable, healthy, helpful in social situations, visually appealing, fun, and high tech"	1–5	Control (food ad) vs combined experimental (e cig ads)
Tarabashkina,[66] 2016	Y	Product evaluation – "Do you think this food (that is, biscuit 1) is tasty, healthy, and could make you popular among other children?" Dichotomous questions.	0–3	Control (toy ad) versus food ad
van Reijmersdal,[71] 2010	Y	Brand image - "I think [bank name] is … " followed by 13 different characteristics, including friendly, modern, dedicated, and trendy, on a scale ranging from 1 (completely disagree) to 7 (completely agree).	1–7	Control (game with no placement) versus combined experimental
Verhellen,[73] 2014	Y	Brand attitude – 3 qs (ie, "I like …,"" … popsicles taste good," and " … popsicles are fun") on a 4 point smiley scale. Calculated a summated scale.	1–4	Control (no ad exposure) versus combined experimental
Petrescu,[64] 2017	Y	Appeal of using e-cigarettes – 3 bipolar items: unattractive versus attractive, not cool versus cool and boring versus fun. Responses were recorded on scales ranging from 1 to 5, with higher scores denoting greater appeal.	1–5	Control (no ads) vs combined experimental conditions of e-cigarette adverts (glamor or health)
Royne,[38] 2017	Y	Attitude - how much they liked each of the 3 drinks tested, how healthy they perceived each of the drinks, 5-point, facial recognition scale; kept as individual data points	1–5	Control (SpongeBob clip with no placement) vs experimental conditions with placements (juice, milk, cola)
